# One-Step Detection of Vancomycin in Whole Blood Using the Lateral Flow Immunoassay

**DOI:** 10.3390/bios14030129

**Published:** 2024-02-29

**Authors:** Yugyung Jung, Seonjong Kim, Min-Gon Kim, Young-Eun Lee, Myung-Geun Shin, Sung Yang

**Affiliations:** 1 Department of Biomedical Science and Engineering, Gwangju Institute of Science and Technology (GIST), Gwangju 61005, Republic of Korea; jyg4789@gist.ac.kr; 2 Department of Chemistry, Gwangju Institute of Science and Technology (GIST), Gwangju 61005, Republic of Korea; mikube@gm.gist.ac.kr (S.K.); mkim@gist.ac.kr (M.-G.K.); 3 GMD Biotech, Inc., Gwangju 61005, Republic of Korea; 4 Department of Laboratory Medicine, Chonnam National University Hwasun Hospital (CNUHH), Hwasun 58128, Republic of Korea; yeun.lee@cnuhh-app.or.kr (Y.-E.L.); mgshin@chonnam.ac.kr (M.-G.S.); 5 Accelerator Platform of Precision Medicine, Chonnam National University Hwasun Hospital (CNUHH), Hwasun 58128, Republic of Korea; 6 School of Mechanical Engineering, Gwangju Institute of Science and Technology (GIST), Gwangju 61005, Republic of Korea

**Keywords:** vancomycin, lateral flow immunoassay, whole blood

## Abstract

Vancomycin (VAN) is an effective antibiotic against Gram-positive bacteria and the first-line therapy to prevent and treat methicillin-resistant *Staphylococcus aureus* (MRSA) and severe infections. However, low concentrations of VAN can result in resistant strains. High doses of VAN can cause nephrotoxicity and ototoxicity; thus, VAN is a representative drug for which drug monitoring is recommended. Several methods have been proposed to detect VAN. Among them, lateral flow immunoassays (LFIAs) have advantages, such as simple and user-friendly operation, low sample volume requirement, and cost effectiveness. In this study, we developed an LFIA capable of rapid on-site detection such that the VAN concentration in plasma could be monitored within 20 min by a one-step detection process using whole blood without plasma separation. VAN can be detected in whole blood over a wide range of concentrations (20−10,000 ng/mL), and the LFIA reported here has a detection limit of 18 ng/mL. The applicability of the developed LFIA compared to the results of measuring VAN with a commercial enzyme-linked immunosorbent assay kit showed a satisfactory correlation (Spearman’s rho, ρ = 0.891). Therefore, the developed LFIA enables rapid and wide-range VAN detection in whole blood and can aid in drug monitoring to evaluate patients’ responses to treatment.

## 1. Introduction

Vancomycin (VAN) is an extensively used glycopeptide-based antibiotic and exhibits antimicrobial activity by inhibiting the cell wall synthesis of Gram-positive bacteria [1,2]. VAN is effective against Gram-positive infections and is used to treat and prevent infections caused by methicillin-resistant *Staphylococcus aureus* (MRSA), a notorious multidrug-resistant super-bacteria associated with hospital-acquired infections. Since the 1980s, as the number of MRSA infections has increased, VAN has been used as a first-line therapy and empirical antibiotic for managing severe infections [1,3,4,5,6,7]. However, an inadequate dose of VAN may result in the development of resistant strains, and a dose above the therapeutic level may cause side effects such as nephrotoxicity and ototoxicity [8,9]. Therefore, VAN is a representative drug recommended for therapeutic drug monitoring (TDM) to minimize side effects and ensure successful treatment results [10]. TDM measures the drug concentration in the blood or plasma at a specified time to maintain a normal range of the drug concentration in the body. This measured drug concentration is used to interpret pharmacokinetic parameters according to the measured values to draw appropriate conclusions about drug concentration and dose adjustment [11]. Although clinical treatment guidelines are provided for general VAN doses, more cautious dose administration is required for high-risk groups such as pediatric patients, patients with kidney failure, and severely ill patients [12,13,14,15]. Moreover, if the drug concentration does not reach the ideal range owing to individual differences in drug metabolism, treatment failure and drug resistance can occur. Therefore, continuous and immediate TDM is required for precise VAN treatment according to individual patient differences, which can reduce the drug’s side effects and medical costs during treatment and provide clinical data for accurately adjusting protocols according to patient disease cases [16].

Methods used for monitoring VAN include high-performance liquid chromatography (HPLC), liquid chromatography/mass spectrometry (LC-MS), and capillary electrophoresis [17,18,19]. These methods provide quantitative data with high sensitivity and accuracy; however, they require sample preparation before analysis and trained technicians to carry out complicated procedures on these expensive types of equipment. Similarly, an enzyme-linked immunosorbent assay (ELISA) method is commonly used at the laboratory scale. ELISAs are not easily accessible to general users because of their complex procedures, involving incubation and washing [20]. Hence, it is difficult for these methods to provide efficient and rapid analysis results for the timely re-adjustment of drugs. Compared to the abovementioned methods, lateral flow immunoassays (LFIAs) use the immune response and chromatography and is commonly used for point-of-care diagnosis. LFIAs have advantages such as its simple procedure, time efficiency, low cost, easy interpretation of results, and a wide detection range [21,22]. Therefore, various studies have been conducted to detect VAN using LFIAs, providing reliable detection results from samples such as milk, serum, and plasma, demonstrating the outstanding advantages of LFIAs [23,24,25]. However, the LFIA method used in these studies had limitations, such as requiring multiple steps during sample preparation or detection. Kong et al. detected VAN in milk and animal feed samples. The method employed in this study involved a two-step detection process: (a) incubating gold nanoparticles (AuNPs) with the target sample and (b) loading it onto the strip [23]. Bian and Liang reported VAN detection in serum samples. For this serum separation, blood had to be pre-treated to remove blood cells and fibrinogen, and separate activation equipment was needed to obtain the fluorescent signal [24]. In addition, Shen et al. reported VAN detection in plasma samples. For the plasma sample, pre-treatment was required to separate blood cells from blood samples, and before using strips, VAN antibodies were fluorescently labeled, and the fluorescently labeled VAN antibody (Ab) served as the detection solution. The detection solution was subsequently mixed with a diluted plasma sample, and then the mixed solution was loaded onto the strip, requiring a two-step detection process for the strip [25]. These procedures make the whole detection process complex, requiring supplementary equipment and extended time for analysis.

The separation of plasma from whole blood for blood tests is one of the procedures required to prevent interference in several clinical diagnostic assays. Following the recent development of microtechnology, numerous studies have been conducted to enable the miniaturization of biochemical processes [26]. By applying electric, magnetic, and acoustic energy to microfluidic technology, plasma separation can be performed with a small blood volume, and different functions can be integrated and miniaturized [27,28,29]. These technologies require a pump or other energy sources for flow; thus, manufacturing and operations may be constrained. However, paper-based separation methods do not require an external driving energy source and are suitable for point-of-care diagnostics that can be directly integrated into LFIA [30,31]. Al-Tamimi et al. introduced plasma separation using red blood cell (RBC) aggregation by treating paper with an anti-H agglutinating monoclonal Ab. They demonstrated the performance of rapid and straightforward paper-based plasma separation from all blood samples [32]. This method used anti-H antigen Ab; it does not impact on the stability of the gold nanoparticle (AuNP) used in the LFIA and can respond to all blood types except the Bombay blood group. However, this study mainly focused on the fundamental plasma separation process without addressing the performance and applicability of the separation method in sensor applications. Additionally, H antigen is a precursor of A/B antigen and is converted into A or B antigen depending on the blood type. It is present in the greatest amount in type O and the smallest amount in type AB. Therefore, the H antigen content of RBCs varies depending on the ABO group and can affect the strength of the reaction when assessed by an agglutination reaction with an anti-H antigen Ab [33,34,35]. Therefore, considering the limitations that may arise due to the characteristics of the H antigen, we attempted to increase the performance of the separation method by using an anti-RBC Ab that can bind to a variety of epitopes not limited to the H antigen.

In the present study, we aimed to use a gold nanoparticle (AuNP)-based competitive LFIA method to detect VAN in whole blood. Detecting small molecules in whole blood using an LFIA requires sample purification due to the complex nature of the blood matrix. The sample pad was treated with cost-effective anti-RBC polyclonal antibodies for plasma separation, which exhibit high affinity by binding to various epitopes. Thus, the objective of this study was to propose an LFIA method that can rapidly and efficiently detect VAN concentrations in a small amount of whole blood in one step without requiring sample pre-treatment or fluorescence signal activation. We anticipate the developed LFIA strip to be an effective point-of-care diagnostic sensor in situations requiring rapid monitoring and diagnosis.

## 2. Materials and Methods

### 2.1. Chemicals and Materials

Whatman Immunopore RP membranes and Grade 5 filter paper were purchased from Cytiva (Marlborough, MA, USA). The absorbent (Grade 222), conjugate (Grade 6613), and sample pads (Grade 8964), Cytosep^®^ (1662) were purchased from Ahlstrom-Munksjö (Helsinki, Finland). Vivid plasma separation GR membrane was purchased from Pall Life Sciences (East Hills, NY, USA). AuNPs (BBGC.40) were purchased from Bore Da Biotech Co., Ltd. (Seongnam-si, Republic of Korea). Tween^®^ 20, D-(+)-trehalose dehydrate, rabbit anti-goat IgG antibody, goat anti-mouse IgG antibody, vancomycin, and teicoplanin were purchased from Sigma-Aldrich Co. (St. Louis, MO, USA). Vancomycin-BSA was purchased from CalBioreagents (San Mateo, CA, USA). The mouse anti-vancomycin antibody (clone 30, CABT-LH054, 1 mg), a purified IgG type, along with the human vancomycin ELISA kit (DEIANJ11) that operates based on the indirect competitive assay principle for human serum and plasma samples, were acquired from Creative Diagnostics (Shirley, NY, USA). The anti-human RBC antibody was purchased from Rockland Immunochemicals (Limerick, PA, USA). Bovine serum albumin (BSA) was purchased from Bovogen Biologicals (Melbourne, Australia). Phosphate-buffered saline (1× PBS, pH 7.4), borate buffer, gentamicin, and H antigen monoclonal antibody were purchased from Thermo Fisher Scientific (Waltham, MD, USA). Cefotaxime and ceftriaxone were purchased from Selleck Chemicals (Houston, TX, USA).

### 2.2. Preparation of Ab-Conjugated AuNP

An anti-mouse IgG (mIgG) Ab was conjugated to the surface of AuNPs via electrostatic adsorption [36]. One milliliter of AuNPs (dia. 40 nm) was mixed with 100 µL borate buffer (0.1 M, pH 8.5). Ten microliters of mIgG Ab (1 mg/mL) were added to the AuNP mixture and incubated for 1 h at room temperature (RT). After incubation, 10 µL of 10% BSA was added and incubated for 1 h to block the AuNP surface. After 1 h, the AuNP mixture was centrifuged at 9000 rpm and 10 °C for 15 min. The supernatant was removed, and the Ab-conjugated AuNP (Ab-AuNP) pellet was suspended in 1 mL borate buffer (10 mM, pH 8.5). This washing step was repeated three times, and in the final washing step, the Ab-AuNPs were re-suspended in 100 µL of 10 mM borate buffer.

### 2.3. Fabrication of an LFIA Strip and Experimental Procedure

**Figure 1:**
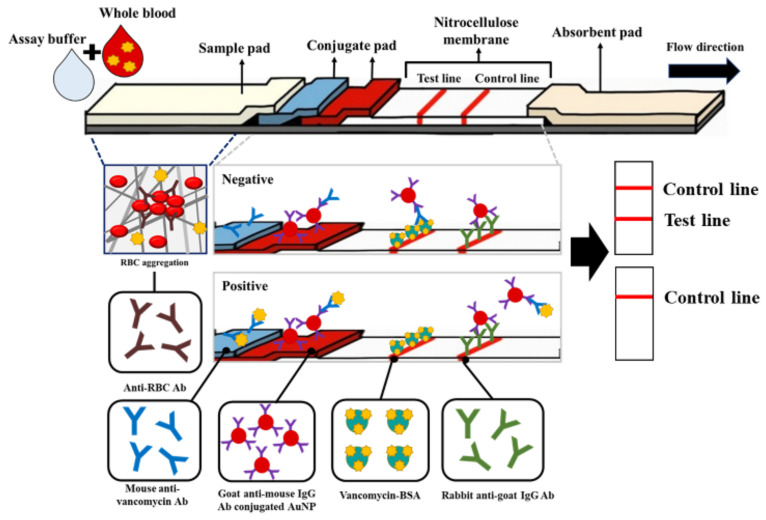
Schematic for the competitive LFIA to detect VAN in whole blood. The lateral flow strip schematic consists of a sample pad, two conjugate pads containing AuNP-conjugated mIgG antibody and VAN antibody, an NC membrane with the test and control lines, and an absorbent pad. Schematic depiction of the principal for the negative or positive reaction depending on VAN. The test and control lines were red for negative VAN.

The LFIA strip consists of a sample pad containing an RBC-Ab, conjugate pads containing the VAN Ab and Ab-AuNPs, a nitrocellulose (NC) membrane containing test and control lines, and an absorbent pad (Figure 1). First, the lines were formed with 500 µg/mL VAN-BSA as a test line and 250 µg/mL anti-goat IgG Ab as a control line on an NC membrane (3.8 × 25 mm) using a dispenser (1 µL/cm). The distance between the test and control lines was approximately 5 mm. The NC membrane was dried at 37 °C for 20 min. The sample pad was treated with RBC Ab for plasma separation. The RBC-Ab treatment conditions of the sample pad were optimal for 10 µL with a concentration of 10 mg/mL (Figures S1 and S2). A plasma separation experiment was also conducted using the H antigen Ab, mentioned in the introduction, and RBC Ab. Based on these results, the RBC Ab showed an improved plasma separation performance and was applied to the sample pad (Figures S3 and S4). After treatment, the sample pad was dried. The conjugate pads (3.8 × 4 mm) were treated with pre-treatment buffer (5% trehalose, 0.5% BSA, 0.2% Tween-20 in 1× PBS) and dried. Thereafter, 3.8 µL of 200 µg/mL VAN Ab and 10× Ab-AuNP were dried on the conjugated pad. For the VAN Ab concentration condition, the detection experiment was conducted at a high concentration of VAN and showed a decreasing trend in intensity at 200 µg/mL of VAN Ab condition (Figure S5). After all the pads were adequately dried, the absorbent pad was overlapped and attached to the NC membrane. A conjugate pad containing the Ab-AuNPs was superimposed on the other side of the absorbent pad. Thereafter, the conjugate pad containing the VAN Ab and the sample pad were sequentially overlapped. Finally, the LFIA strip assembly was completed. The assembled LFIA was inserted into a microwell plate containing a 1:9 mixture of 5 µL of sample and 45 µL of assay buffer (PBST 0.1%) for 20 min to conduct the assay (Figure S6). Thereafter, images of the test and control lines were obtained using a ChemiDoc XPS (Bio-Rad, Hercules, CA, USA), and line intensities were measured using Image Lab software (ver 6.1, Bio-Rad).

### 2.4. Detection Principle of the Competitive LFIA

The competitive LFIA is primarily used to detect small molecules [37]. The detection principle is based on a competitive binding reaction between the target in the sample and the target immobilized on the test line of the NC membrane. When the sample to be detected is mixed with the assay buffer and loaded onto the sample pad, the mixture moves toward the absorbent pad via capillary force. In the absence of VAN, the VAN Ab of the conjugate pad migrates to the membrane and binds to its test line competitor (VAN-BSA). AuNP-conjugated antibodies can specifically bind to mouse IgG bound to the VAN Ab. Therefore, this reaction forms a red line on the test line. The remaining AuNPs that do not bind to the VAN Ab move toward the control line and bind to the rabbit anti-goat IgG Ab that captures the goat Ab. A red line is also formed on the control line. In contrast, when VAN is present in the sample, the VAN Ab in the conjugated pad moves to the NC membrane in a state bound to VAN. The VAN Ab, combined with VAN, migrates to the absorbent pad without binding to the VAN-BSA of the test line. Therefore, Ab-AuNPs also pass without binding to the test line because of the absence of the VAN Ab. Consequently, the test line is not visible. However, it binds to the anti-goat IgG Ab in the control line, forming a red line. This process was performed in accordance with that reported in a previous study [38].

### 2.5. Preparation of the VAN-Spiked Blood Sample

This study used blood samples supplied only for research after obtaining approval from the Institutional Review Board (CNUHH-2023-154). Before use, blood was stored at 4 °C. The VAN stock solution was serially diluted to 1, 10, 100, 1000, 10,000, 100,000, and 1,000,000 ng/mL in the whole blood. Each sample (5 µL) was absorbed into the LFIA along with 45 µL of assay buffer. For pre-treated samples using centrifugation, whole blood and assay buffer were mixed in a 1:9 ratio, centrifuged at 3000 rpm for 15 min at RT, and only the supernatant was collected and loaded onto the LFIA.

### 2.6. Preparation of Blood Sample with Various Hematocrit (Hct) Levels

Whole blood from the same donor was centrifuged at 3000 rpm for 15 min, and the plasma and blood cells were separated. The isolated blood cells were remixed with plasma according to 35, 45, and 55% Hct levels. The Hct of the mixed blood was measured using a micro-hematocrit centrifuge (HA-200, Hanil Science Medical, Daejeon, Republic of Korea).

### 2.7. Statistical Analyses

All statistical analyses were performed using IBM SPSS statistics 29.0.1.0 (demo version; IBM Corp., SPSS Inc., Armonk, NY, USA). A *p*-value < 0.05 was considered statistically significant. Graphs and logistic fitting were prepared using OriginLab 2021 (OriginLab^®^, Northampton, MA, USA).

## 3. Results and Discussion

### 3.1. Evaluation of the RBC-Ab-Treated LFIA with Variation in Hct Levels

**Figure 2:**
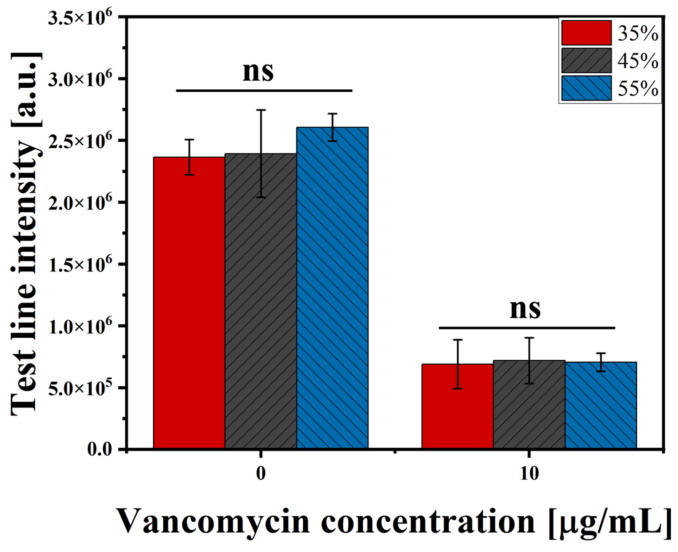
VAN detection using RBC-Ab-treated LFIA in test line with various Hct values. Under the absence and presence of VAN (0, 10 µg/mL), test lines were measured at 35%, 45%, and 55% of Hct. Each error bar represents the standard deviation of four measurements. ns: not significant.

The test line of the RBC-Ab-treated LFIA was measured in blood samples with three different Hct levels. Hct is an important factor that affects blood separation, and the higher the value is, the greater the proportion of RBCs in the whole blood, which can cause interference in detection. The Hct normal range is 37−48% in women and 42−52% in men. Therefore, three blood samples with Hct levels of 35, 45, and 55% were prepared to evaluate plasma separation according to Hct, under optimal conditions of RBC antibodies. The test line intensity values of each of the three samples were 2.36 × 10^6^, 2.39 × 10^6^, and 2.61 × 10^6^ a.u. under the 0 µg/mL VAN concentration. They were 6.89 × 10^5^, 7.18 × 10^5^, and 7.05 × 10^5^ a.u. under the 10 µg/mL of VAN concentration, respectively (Figure 2). According to the Kruskal-Wallis test, the *p*-value was 0.246 at 0 µg/mL VAN and 0.944 at 10 µg/mL VAN, confirming that there is no significant difference in the intensity values of the test lines according to the 35, 45, and 55% Hct conditions. This finding indicated that there is no difference in the RBC removal efficiency according to the Hct levels.

### 3.2. Evaluation of RBC-Ab-Treated LFIA

**Figure 3:**
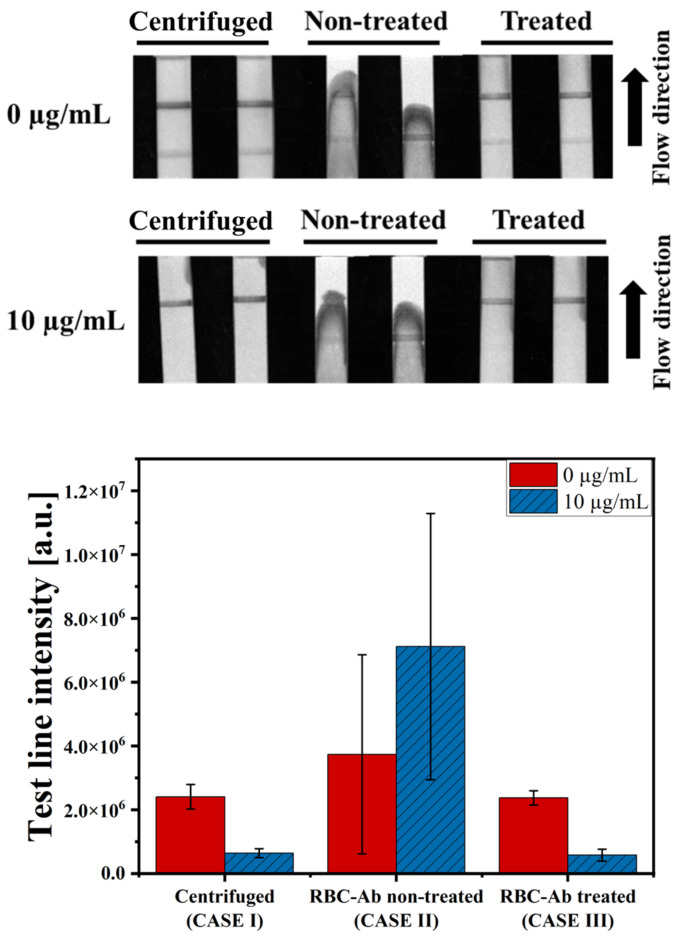
Comparison of the LFIA results. CASE I: centrifuged whole blood with RBC-Ab non-treated LFIA, CASE II: whole blood with RBC-Ab non-treated LFIA, and CASE III: whole blood with RBC-Ab-treated LFIA. In all cases, blood Hct was 55%. Each error bar represents the standard deviation of four measurements.

The test line intensity was compared for the three cases to verify the performance of the RBC-Ab-treated LFIA. In the first case (CASE I), the LFIA without the RBC-Ab treatment was carried out with the blood plasma harvested via centrifugation of whole blood. In the second case (CASE II), the LFIA without the RBC-Ab treatment was carried out with whole blood. In the third case (CASE III), the LFIA with RBC-Ab treatment was carried out with whole blood. The whole blood sample used in the experiment was diluted with the assay buffer at a ratio of 1:9 before loading on the strip. In CASE I, centrifugation was performed after a 1:9 dilution, and the supernatant (diluted blood plasma) was used for the LFIA. All assay times were maintained at 20 min. As shown in Figure 3, the test line intensity of CASE I and CASE III had values of 2.44 × 10^6^ and 2.46 × 10^6^ a.u. under the 0 µg/mL VAN condition, and 6.73 × 10^5^ and 6.31 × 10^5^ a.u. under the 10 µg/mL VAN condition, respectively. According to the Mann-Whitney U test, the *p*-value was 1.00 for 0 µg/mL and 10 µg/mL conditions, showing no statistically significant difference. In contrast, in CASE II, blood cells were clogged on the NC membrane, as shown in the strip image in Figure 3; thus, the sample mixture did not reach the absorbent pad and stopped at the NC membrane. Consequently, due to the influence of blood cells in the test line, the average intensity value showed a markedly greater magnitude than the others, and the standard deviations were also large (0 µg/mL: 9.17 × 10^6^ ± 8.73 × 10^6^ a.u., 10 µg/mL: 4.95 × 10^6^ ± 4.02 × 10^6^ a.u.). This result indicates that the LFIA treated with RBC-Ab can detect VAN with a performance similar to that under centrifuge conditions by removing RBCs that influence VAN detection in the test line. In addition, comparative experiments were conducted with paper-based commercial products for plasma separation (Figure S7).

### 3.3. Detection of VAN in Human Whole Blood

**Figure 4:**
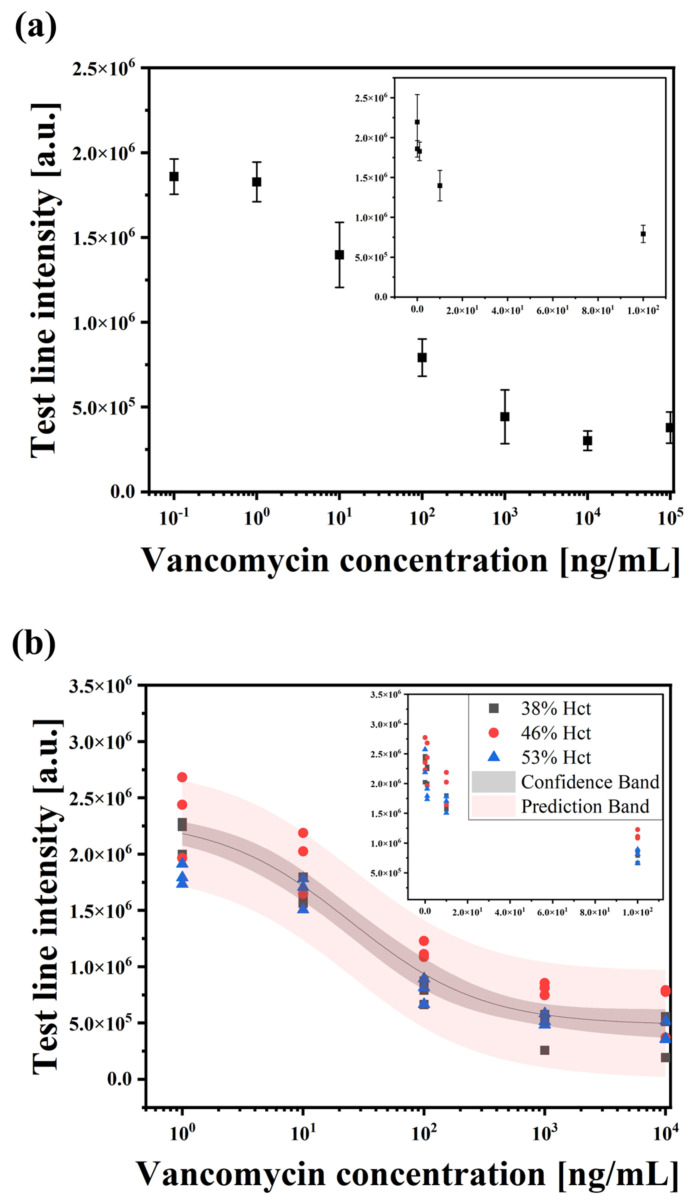
Analytical sensitivity of the developed LFIA for VAN detection in whole blood. (**a**) The calibration curve of the test line was drawn in the 0.1 ng/mL−100 µg/mL range. Each error bar represents the standard deviation of four measurements. (**b**) VAN (1−10 µg/mL) detection with three different blood samples. The confidence interval and the prediction interval were calculated with a 95% confidence level. Each sample was measured three times. The inset shows the linear range of VAN concentration from 0 to 100 ng/mL.

To evaluate the analytical performance of the developed LFIA, VAN was spiked in whole blood. Eight samples with different VAN concentrations (0, 1, 10, 100, 1000, 10,000, 100,000, and 1,000,000 ng/mL) were prepared using the assay buffer to investigate the detection range of VAN. As shown in Figure 4a, as the concentration of VAN in the sample increases, the binding between VAN Ab and VAN-BSA on the test line diminishes, resulting in a decrease in the intensity of the test line (1−10,000 ng/mL). The intensities of the test lines at low concentrations (0.1 and 1 ng/mL) did not differ significantly (*p*-value = 0.886). The intensity tended to increase at the high concentration of 100,000 ng/mL, which may be caused by the hook effect [39]. Three blood samples were used to obtain the test line intensity values based on the VAN concentration of each sample. The performance of the LFIA was also treated with respect to the level of Hct (38, 46, and 53%). As shown in Figure 4b, the test line intensities decrease as the VAN concentrations increase in the three samples, as expected. The coefficient of variation (CV) values were calculated to indicate repeatability, which can be found in Table S1. Data points beyond the confidence interval have been observed. The results’ deviations from the model predictions may be attributed to specific variability or exceptional conditions, given that confidence intervals are for the population mean rather than individual values. Meanwhile, data that deviated from the prediction interval were extremely limited. The graphs show R^2^ values of 0.999, 0.994, and 0.958 for each sample, based on logistic fitting. The detection limit of the LFIA for whole blood samples was 18 ng/mL (3 × SD blank/slope), and the detection range was 20−10,000 ng/mL. The slope was obtained from a linear range from 0 to 100 ng/mL (R^2^ = 0.802).

### 3.4. Selectivity Test for VAN

**Figure 5:**
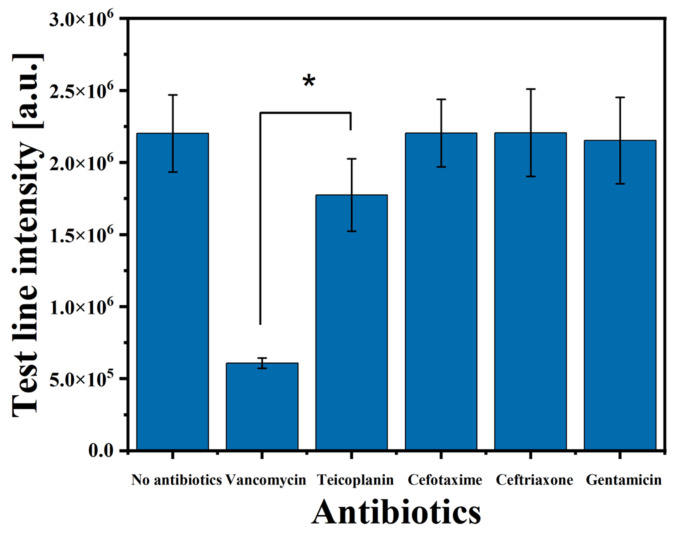
Selectivity experiment for VAN against other antibiotics at the same concentration of 100 µg/mL in whole blood. Each error bar represents the standard deviation of four measurements. One asterisk (*) indicates a statistical significance of *p* < 0.05, respectively.

The selectivity evaluation of the LFIA strip was confirmed using a cross-reactivity test. The LFIA was assessed against the third-generation cephalosporin antibiotics that can be used with VAN (cefotaxime, ceftriaxone), the aminoglycoside (gentamicin), and the teicoplanin (a glycopeptide antibiotic with a structure similar to that of VAN). Each antibiotic was spiked independently in whole blood at a 100 µg/mL fixed concentration. As shown in Figure 5, the intensity values are obtained by measuring the test line. Most antibiotics show results similar to the negative sample (no antibiotics). Teicoplanin, structurally similar to vancomycin, showed a slightly lower intensity value than the other antibiotics. However, this difference was not statistically significant (*p* = 0.156) but significantly differed from VAN (*p* = 0.029). In contrast, the intensity of the test line was considerably lower in the sample containing VAN. Therefore, it was proven that the LFIA exhibited higher selectivity for VAN.

### 3.5. Comparison with the Conventional Method, ELISA

**Figure 6:**
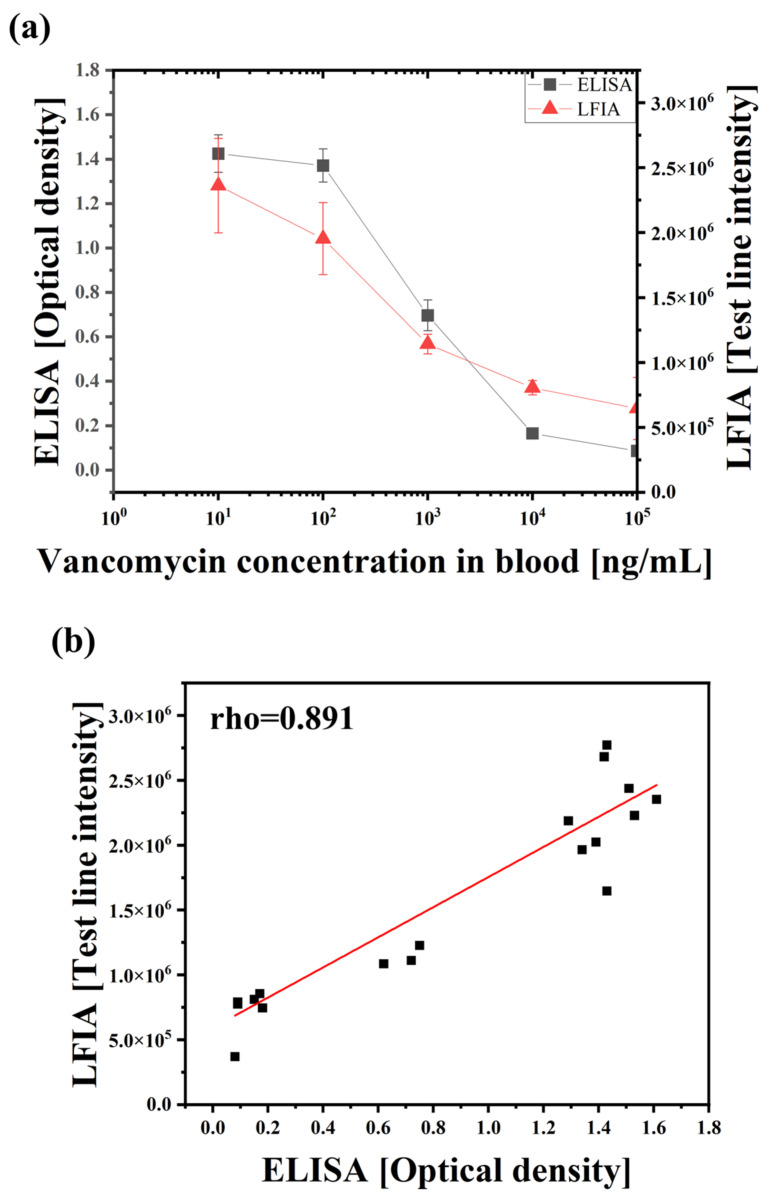
Comparison of the developed LFIA and commercial ELISA kit. (**a**) LFIA and ELISA calibration curves according to VAN concentration (0, 10, 100, 1000, 10,000, and 100,000 ng/mL in blood). Each error bar represents the standard deviation of three measurements. (**b**) Correlation analysis between the ELISA and developed LFIA. Data were analyzed using Spearman’s rank correlation, rho = 0.891, *p* < 0.001. The LFIA intensity data are the same data measured on the 46% Hct sample mentioned in Figure 4b.

Finally, VAN detection was performed in the same sample using the proposed LFIA and conventional competitive ELISA methods to demonstrate the applicability of the LFIA system. The samples were prepared by spiking blood with VAN. The VAN samples were diluted with assay buffer at a ratio of 1:1000 for the ELISA kit and 1:10 for the LFIA. The VAN detected using the proposed LFIA and commercial VAN ELISA kits is shown in Figure 6a; both the LFIA’s intensity and the ELISA’s optical density decreased as the VAN concentration increased. To analyze the correlation between the two methods, Spearman’s rho, a non-parametric method, was used, and the strength of the correlation can be interpreted as strong when it is 0.8 or higher [40]. As a result of the Spearman analysis, a linear graph was obtained for the two methods, and the correlation coefficient was 0.891 (*p* < 0.001), which was a statistically significant correlation between both assay readings (Figure 6b). This finding showed that the LFIA yielded satisfactory analytical performance and could measure VAN concentrations in whole blood.

## 4. Conclusions

Monitoring VAN, which is widely used as an empirical antibiotic and first-line treatment for severe infections, including MRSA, is important for optimizing the efficacy of the drug and lowering the risk of toxicity, thereby increasing its therapeutic effect. There are several methods for detecting VAN; however, various inconvenient processes are involved in its detection in whole blood. In this study, we developed a one-step LFIA capable of detecting VAN in a small amount of whole blood. The developed LFIA can detect VAN in blood without pre-treatment and can cover a wide range of VAN concentrations, even with a small volume (5 µL), so it is expected to be useful even in situations where only small amounts of blood can be obtained, such as from neonates [41]. To remove RBCs, which account for 99% of total blood cells, the sample pad was treated with an RBC Ab which induces RBC aggregation, preventing RBCs from passing through the membrane, and thereby reducing interference in the test line. This was tested at various Hct levels, and it was verified that there was no significant difference in the test line results. The proposed LFIA was able to detect VAN concentrations ranging from 20 ng/mL to 10,000 ng/mL within 20 min, using three whole blood samples spiked with VAN. The performance of the sensor was quantified using an imaging program, and it showed a detection limit of 18 ng/mL. By comparing the intensity value of the LFIA according to the VAN concentration with the optical density measured using a commercial ELISA kit, which is a conventional method, the Spearman correlation efficiency was 0.891. This result showed that the proposed sensor effectively detects VAN in whole blood. Therefore, the proposed LFIA can be applied to VAN and other drugs that require blood drug monitoring.

The experiments thus far have shown the methodological verification of a one-step LFIA developed using spiked VAN blood samples. However, the performance of the sensor could not be verified in the blood of patients prescribed VAN; therefore, additional clinical verification is required. The next step is to compare the clinical validation of the developed LFIA in actual patient samples with the TDM measurement method used in actual clinical practice, considering the protein binding characteristics of VAN in the blood, through a collaborative study with local hospitals.

## Data Availability

Data are contained within the article.

## Supplementary Materials

The following supporting information can be downloaded at https://www.mdpi.com/article/10.3390/bios14030129/s1, Figure S1: Optimization of anti-RBC antibody conditions on the consistently sized sample pad; Figure S2: Optimization of anti-RBC antibody conditions, evaluating the test and control lines; Figure S3: Preliminary test with H antigen and RBC antibody; Figure S4: VAN detection according to blood type using LFIA treated with anti-RBC antibody; Figure S5: Optimization of anti-VAN antibody conditions; Figure S6: Illustration of the dipstick assay; Figure S7: Comparison with paper-based commercial products. Table S1: Coefficient of variation (CV) of three samples according to hematocrit levels.
