# Supplementary material for: One-Step Detection of Vancomycin in Whole Blood Using the Lateral Flow Immunoassay
Source: Biosensors (Basel). 2024 Feb 29;14(3):129. doi: 10.3390/bios14030129 (PMC10968287; doi:10.3390/bios14030129)
Supplement: Supplementary file 1 [file biosensors-14-00129-s001.zip › biosensors-2831764-supplementary.pdf]

biosensors

Supplementary materials

One-step detection of vancomycin in whole blood using the lateral flow immunoassay

Yugyung Jung [1], Seonjong Kim [2], Min-Gon Kim [2,3], Young-Eun Lee [4,5], Myung-Geun Shin [4,5], and Sung Yang [1,6,*]

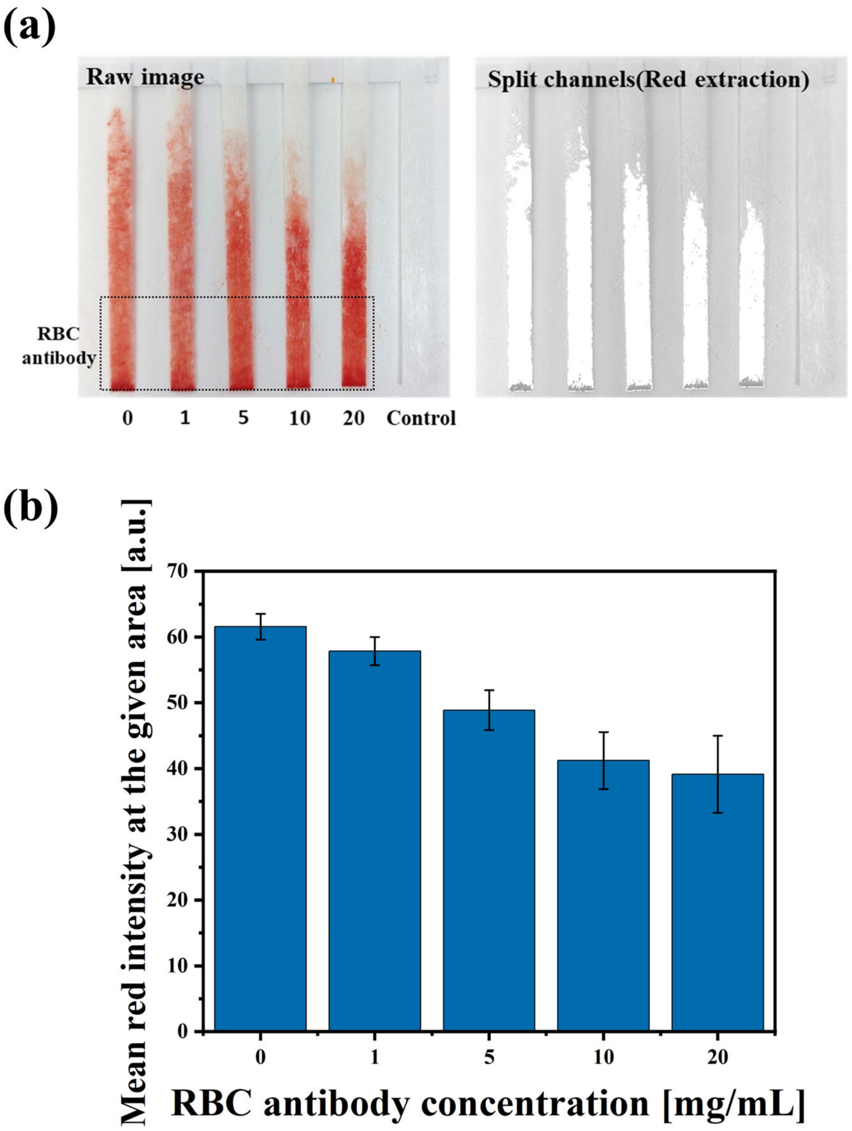

Figure S1. Optimization of anti-RBC antibody condition on the consistent-sized sample pad. The image of the sample pads obtained by loading the whole blood mixed with the assay buffer in a 1:9 ratio on the sample pad treated with 10 µL of various RBC antibody concentrations (0, 1, 5, 10, 20 mg/mL) was analyzed. (a) Image of the sample pad by the treated concentration of RBC antibody. The Image J free software (NIH, Bethesda, MD, USA) extracted the red color from the raw image using the split channel function. (b) The red intensity was calculated as the average red value in the entire sample pad area minus the average red value in the control pad without blood. The red intensity value tended to decrease as the RBC antibody concentration increased. Each error bar represents the standard deviation of three measurements.

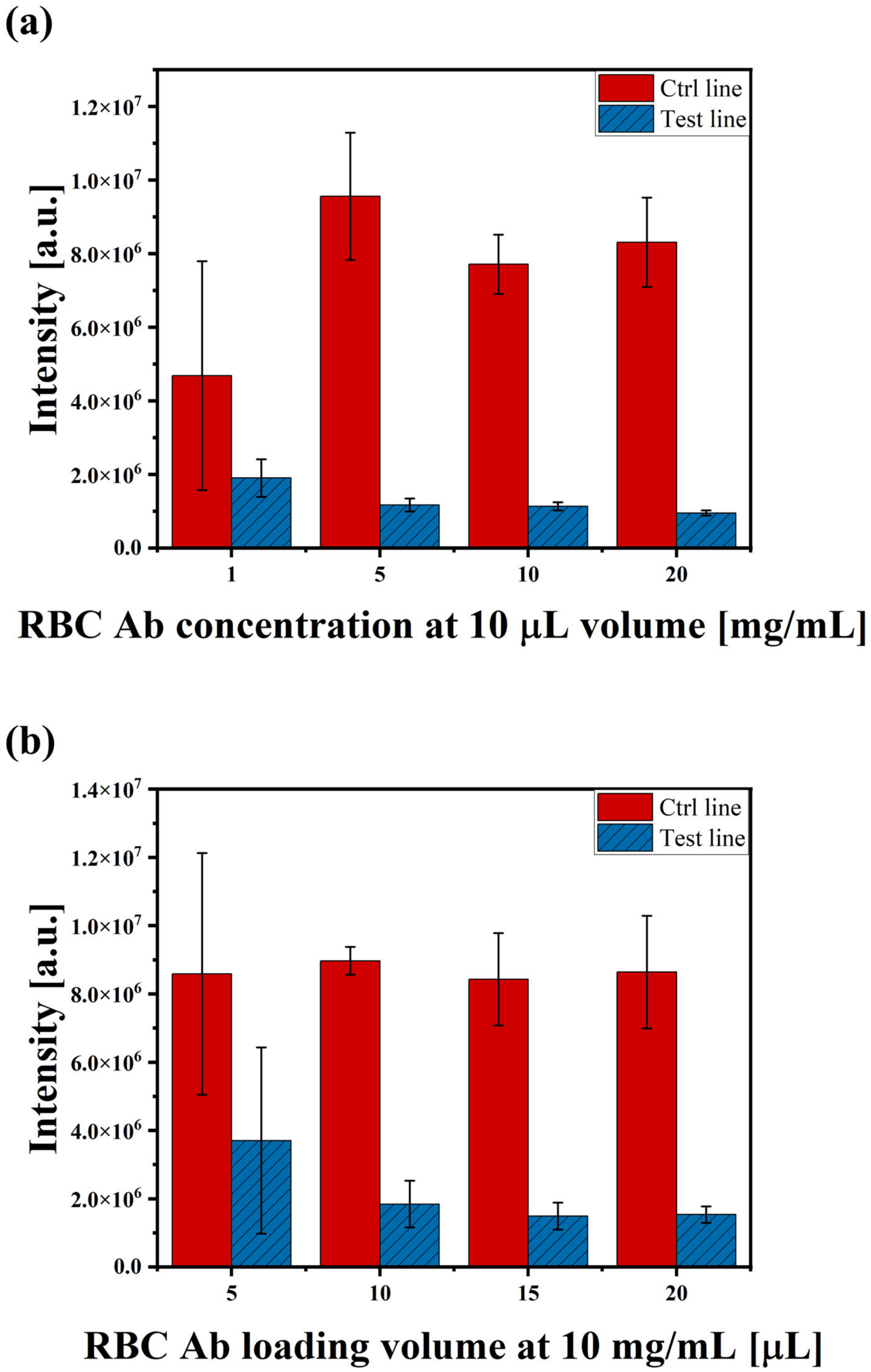

Figure S2. Optimization of anti-RBC antibody condition evaluating with test and control line. (a) Effect of concentration of anti-RBC antibody with different concentrations (1, 5, 10, 20 mg/mL). The standard deviation was lower at 10 mg/mL when the experiment was conducted under the same conditions (10 μL of RBC Ab loading volume). (b) RBC antibodies were applied to sample pads at different loading volumes (5, 10, 15, 20 μL) with a 10 mg/mL concentration. The test and control lines were measured in the absence of VAN. At a volume of 10 μL, both the control and test lines exhibited stable standard deviation values, and the average intensity values were similar at volumes equal to or greater than 10 μL. Each error bar represents the standard deviation of three measurements.

A preliminary experiment was conducted with the H antigen Ab mentioned in the introduction and RBC Ab. An immunoassay was performed to investigate the capture efficiency of H antigen Ab, comparing blood types O (highest H antigen expression) and AB (lowest H antigen expression). First, antibodies were loaded into a 96-well plate at a concentration of 2 μg/mL, and incubation was performed at 4 ℃ for 12 h. After incubation, washing was performed, and the wells were blocked using 1 % BSA at room temperature for 1 h. After washing, one type AB sample and one type O sample were diluted to a similar cell concentration, dispensed into three wells each, and incubated at room temperature for 2 h. After washing, when loading the TMB solution, it reacts with the captured RBCs, and the color of the transparent solution changes to blue. After adding the stop solution, it was measured at a wavelength of 450 nm. As shown in Figure 3a, H antigen Ab treated wells exhibited a higher optical density (O.D) in the case of type O, whereas a lower O.D was observed in the case of type AB. In contrast, wells treated with RBC Ab showed similar O.D values for both blood types O and AB. In conclusion, H antigen antibody appears to demonstrate an impact based on blood type. To confirm the performance on LFIA strip, 10 μL each of H antigen Ab (20 μg/mL) and RBC Ab (10 mg/mL) was treated on grade 5 filter paper and used as a sample pad. The strip consisted of a sample pad, a conjugate pad containing AuNP, a membrane, and an absorbent pad. One type O whole blood sample was diluted 10× with assay buffer and loaded and measured three times. Consequently, the sample flow was affected by the sample pad and could not move to the end of the membrane, and the control line appeared faint and was not constant. Therefore, the experiment was conducted by changing the sample pad to grade 8964. In the case of the strip using RBC Ab, the samples were developed to the end regardless of blood type. However, in the case of H antigen Ab, although the flow was improved, it was observed that some RBCs migrated to the membrane, and the antigen-antibody reaction was not completed at the control line.

(a)

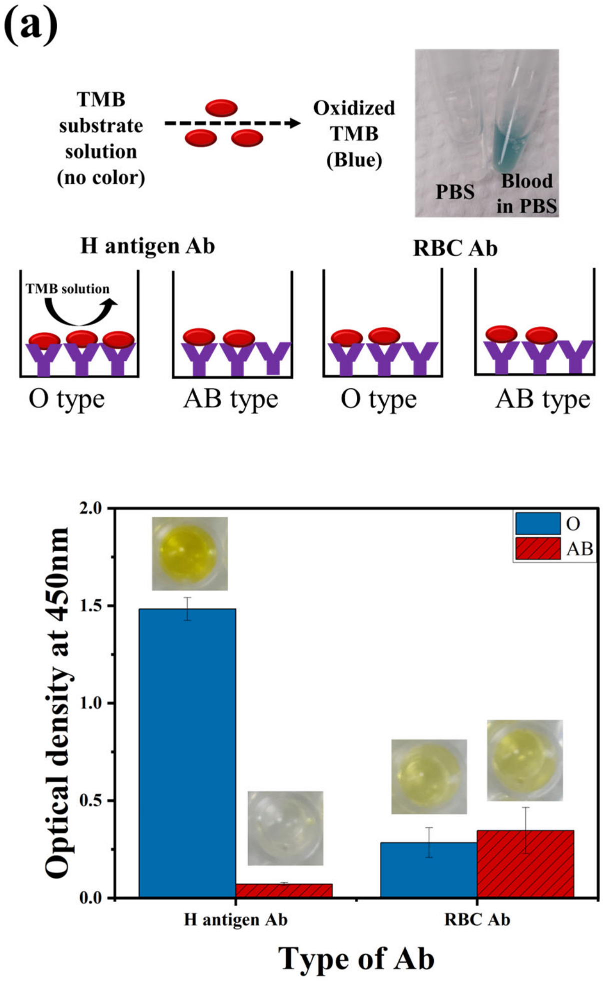

(b)

Condition	H-Antigen Ab	RBC Ab
concentration	20 μg/mL	10 mg/mL
Loading volume	10 μL	
Sample pad	Grade 5	
Blood type	O type	
Assay time	20 min	
Assay (10×Dilution)	45 μL PBST : 5 μL whole blood	

O type

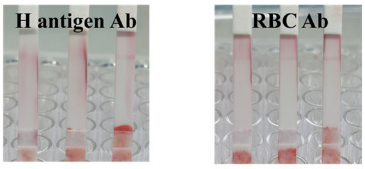

(c)

Condition	H-Antigen Ab	RBC Ab
concentration	20 μg/mL	10 mg/mL
Loading volume	10 μL	
Sample pad	Grade 8964	
Blood type	O type vs. AB type	
Assay time	20 min	
Assay (10×Dilution)	45 μL PBST : 5 μL whole blood	

O type AB type

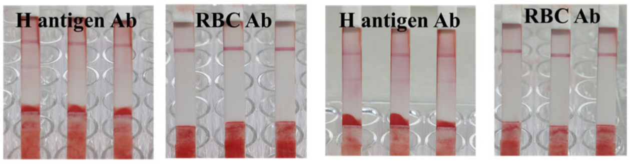

Figure S3. A preliminary test with H antigen and RBC antibody. (a) Immunoassay test for H antigen Ab and RBC Ab according to the blood type O and AB. Each error bar represents the standard deviation of three measurements. (b) H antigen Ab and RBC Ab test on strip using grade 5 sample pad. In both results, the sample flow is affected by the sample pad, impeding its progression to the absorbent pad, and the control line exhibits inconsistent intensity. (c) H antigen Ab and RBC Ab test on strip using grade 8964 sample pad. In contrast to the sample pad treated with RBC Ab, RBCs have risen to the membrane on the sample pad treated with H antigen Ab. The sample containing AuNP is observed to reside at the terminal end of the membrane, and the Antigen-Ab reaction between AuNP and the control line is incompletely resolved.

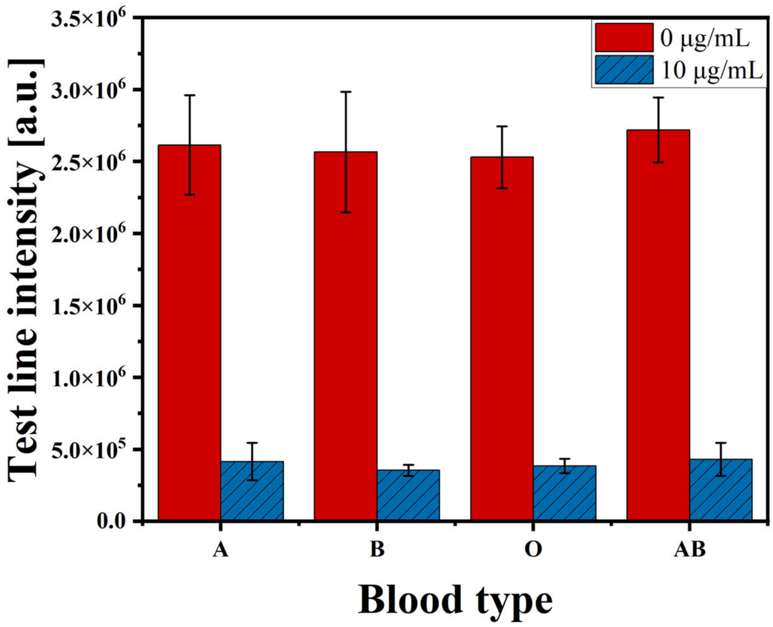

Figure S4. VAN detection according to blood type using LFIA treated with anti-RBC antibody. This result represents those detected at VAN concentrations of 0 μg/mL and 10 μg/mL for each blood type (A, B, O, AB) and was obtained from the intensity of the test line. This finding indicates no difference in RBC removal efficiency depending on blood type. This experiment was conducted using blood from 3 people for each blood type and a total of 12 people.

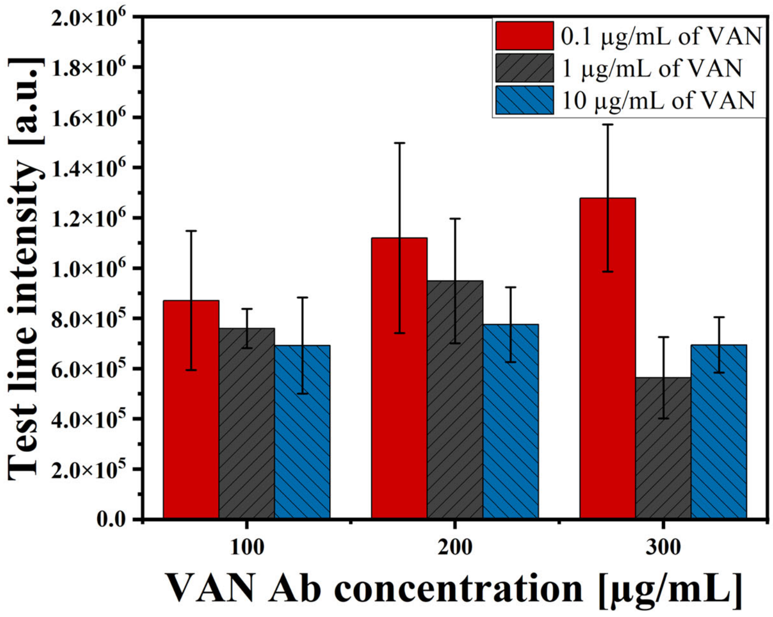

Figure S5. Optimization of anti-VAN antibody condition. The antibody concentration was tested with 0.1, 1, 10 μg/mL of VAN concentration in whole blood. Detection according to the VAN concentration under 200 μg/mL VAN antibody showed the best results. Each error bar represents the standard deviation of three measurements.

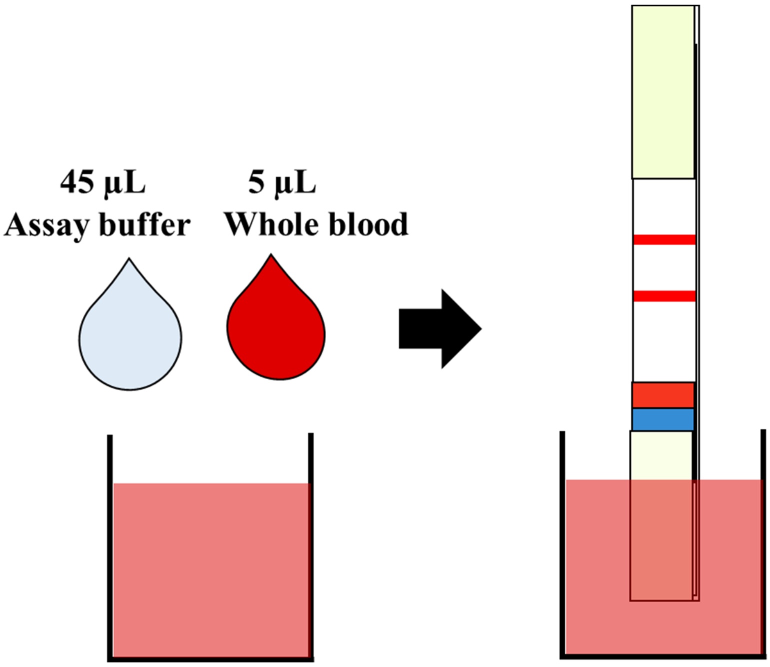

Figure S6. Illustration of the dip-stick assay. All assays were conducted by dipping the LFIA strip into a 96-microwell plate containing 45 μL of assay buffer and 5 μL of the sample.

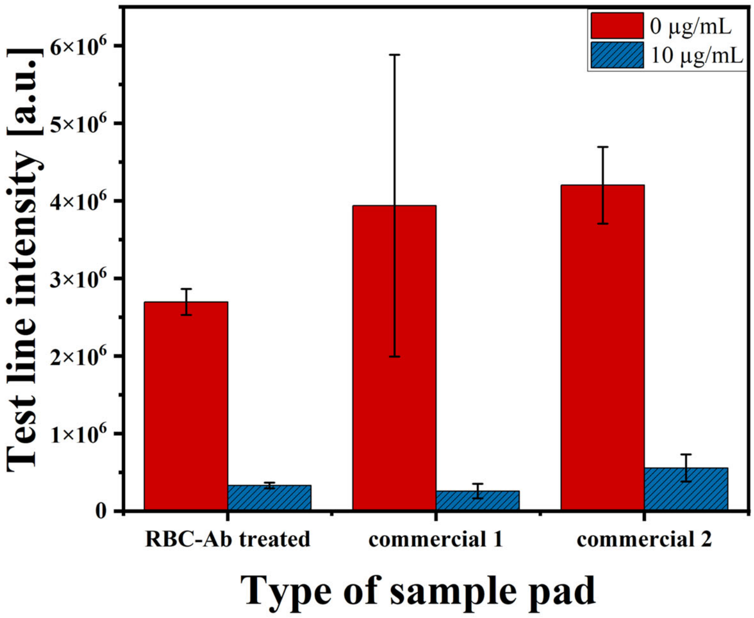

Figure S7. Comparison with paper-based commercial products. Test line intensity results at 0 and 10 μg/mL VAN concentrations in 55% Hct. Although the intensity values are lower than those of the commercial product, the RBC-Ab treated sample pad showed consistent detection with a slight standard deviation. Each error bar represents the standard deviation of three measurements.

Vancomycin	38% Hct	46% Hct	53% Hct
Conc. (ng/mL)	CV (%), n=3		
0	10.3	11.6	9.6
1	7.1	15.4	5.0
10	7.2	14.2	8.5
100	13.5	6.6	14.5
1,000	37.7	6.9	9.8
10,000	47.2	37.0	22.4

Table.S1 Coefficient of variation (CV) of three samples according to hematocrit levels. Table. S1 presents the results of the coefficient of variation (CV) calculations based on the data from Figure. 4b, which includes three repeated measurements for each hct level sample. It was observed that the CV values are higher than expected at certain concentrations, and this variability tends to increase with higher concentrations of vancomycin. This indicates that due to the characteristic of the sensor that the intensity value decreases as the concentration increases, the low intensity value at a concentration of 1000 ng/mL or more is greatly affected in proportion to minute changes in concentration, which means that the CV value may increase as a result.
